# Antioxidant and antidiabetic activities of a polyphenol rich extract obtained from *Abelmoschus esculentus* (okra) seeds using optimized conditions in microwave-assisted extraction (MAE)

**DOI:** 10.3389/fnut.2022.1030385

**Published:** 2022-10-28

**Authors:** Cerile Ypolyte Woumbo, Dieudonné Kuate, Danielle Gaelle Metue Tamo, Hilaire Macaire Womeni

**Affiliations:** Department of Biochemistry, Faculty of Sciences, University of Dschang, Dschang, Cameroon

**Keywords:** optimization, phenol, diabetes mellitus, okra, microwave assisted extraction

## Abstract

Functional foods have gained popularity in recent decades. They are exploited for their bioactive compounds like polyphenols, which are highly demanded in cosmetic, pharmaceutical and nutraceutical industries. However, extractive techniques and conditions used up to recently are almost obsolete and must be optimized for higher efficiency. The current study aimed to evaluate the antidiabetic potential of an optimized extract of *Abelmoschus esculentus* (okra) seeds. The optimal conditions for extracting polyphenolic compounds from okra seeds were determined using Microwave Assisted Extraction (MAE). A Face Center Composite Design (FCCD) was used for optimization. Solvent/dry matter ratio, wavelength and time were considered while the response studied was the polyphenolic content. The extract obtained at optimal conditions was characterized using Thin Layer Chromatography (TLC) and Fourier Transform Infra-Red (FTIR) spectroscopy, then tested for its antioxidant, alpha amylase inhibitory and antidiabetic activities. Response Surface Methodology (RSM) permitted the determination of the optimal conditions for phenols extraction as: microwave power 330 W, with a solvent ratio of 97.04/1 mL/g for 9.5 min of extraction time. The optimized extract showed a phenolic content up to 86.37 ± 1.13 mg GAE/g containing quercetin and catechin as revealed by the TLC. Functional groups characteristic of polyphenols were identified on FTIR spectra, and the extract exhibited good *in vitro* antioxidant capacities with DPPH (2, 2-diphenyl-1-picrylhydrazyl) radical scavenging capacity and FRAP (Ferric Reducing Antioxidant Power Assay). An IC_50_ of 3.99 ± 0.15 μg/mL was obtained with the DPPH scavenging test. Alpha amylase inhibitory assay revealed that the optimized okra extract behaved as a non-competitive inhibitor of porcine pancreatic amylase with an IC_50_ of 484.17 ± 2.33 μg/mL. Antidiabetic activity of the extract was observed in streptozotocin-induced diabetic males Wistar rats, as shown by the fasting blood glucose levels, food intake, changes in body weight and serum lipid profile among others.

## Introduction

Considerable adverse side effects of oral antidiabetics used up to date have reinforced the interest of scientists and patients in functional foods and their derivatives for the management of many chronic diseases (1). *Abelmoschus esculentus* (okra) fruits, for example, have long been investigated for their antidiabetic potential. Seeds, peels or the whole fruits have shown their efficacy in reducing blood glucose levels in experimental animals. Authors mostly attributed the antidiabetic activity of okra fruits and other plants to their polyphenolic content (2). However, the extraction techniques that had been used seem rudimentary compared to what is done nowadays (3). Content of bioactive compounds obtained by traditional extraction methods such as distillation, maceration and solvent extraction among others, used by the previous researchers can be highly improved by cutting-edge techniques like pressurized hot water (PHWE), microwave-assisted (MAE) and ultrasound-assisted (UAE) extractions, thus allowing a potentially great amelioration in the antidiabetic activity of okra fruits or parts. Also the extraction time is significantly reduced by these new techniques (4, 5). Meanwhile each extraction technique has its own advantages and limitations; MAE has demonstrated its high efficiency for a fast extraction of good quality bioactive compounds from natural sources when the solvent is well chosen (6). Okra seeds have been reported as the richest part of the fruits in polyphenols and flavonoids in general, and also in demonstrated antidiabetic compounds such as quercetin and its derivatives in particular (3).

Flavonoids, just like other phenolic compounds are also known to have antioxidants, anti-inflammatory, anti-cancer activities among others (7) and are highly demanded nowadays by cosmetic, pharmaceutical and nutraceutical industries. So, determination of experimental conditions for maximum extraction of polyphenolic compounds from okra seeds using a more efficient technique is quite urgent in order to reduce pollution, solvent and time wastage and above all improve the extraction yield, the quality and the bioactivity of the extracts (8–10). Geng et al. (11) have determined the optimal conditions for extracting polyphenols from okra flowers using conventional maceration technique while Amirabbasi et al. (6) established the conditions for a maximal extraction of polyphenols from okra stems using MAE and UAE. This study aimed to determine the optimal conditions for extraction of polyphenols from okra seeds by a face centered composite design, using MAE and to evaluate the antidiabetic activity of the extract obtained.

## Materials and methods

### Material

#### Plant material

Mature fruits of *Abelmoschus esculentus* were harvested from our botanical garden situated in Dschang (5°27^/^nord, 10° 04^/^ east) West region of Cameroon. The fruits were shade dried and the pods opened for seed collection.

#### Chemicals

All reagents were purchased from local stores while streptozotocin was provided by Sigma-Aldrich (Hamburg; Germany).

#### Experimental animals

To evaluate the antidiabetic activity of the optimized extract, twenty four 24 Wistar rats were collected from the animal house of the department of Biochemistry (University of Dschang) and placed in the animal room of the research unit. For 1 week, rats were fed a normal diet and water for acclimatization. All the animals (after dividing into groups) were housed in individual cages in the animal room under optimal conditions of light (12/12 light and dark cycle), temperature (27 ± 2°C), relative humidity (60 ± 10%) and a pathogen-free surrounding.

### Method

#### Preparation of sample

Seeds of *Abelmoschus esculentus* were dried in an oven at 45°C until constant weight (24 h), and then finely ground using an electrical grinder (royalty line, 800 W, five cycles of 1 min each at full power). Powders were sieved (using a 500 μm sieve) and immediately used for extraction of phenolic compounds.

#### Choosing factors affecting the phenolic content

Based on the literature, the following factors were considered: time of extraction, dry matter/solvent ratio and the power of the microwave oven. A 50:50 hydroethanolic solution was used as solvent system, based on previous works (12, 13). Also, literature indicates that the solvent's dielectric properties should be highly considered when planning to extract phenolic compounds using MAE. Compared to water, ethanol or its mixtures with water have a lower dielectric constant, and are more transparent to microwave, thus not well converting microwaves into heat, but have high capacity to dissolve and extract phenolic compounds (14). Preliminary studies permitted to determine the ranges used for each factors.

#### Determination of responses

**Figure 1:**
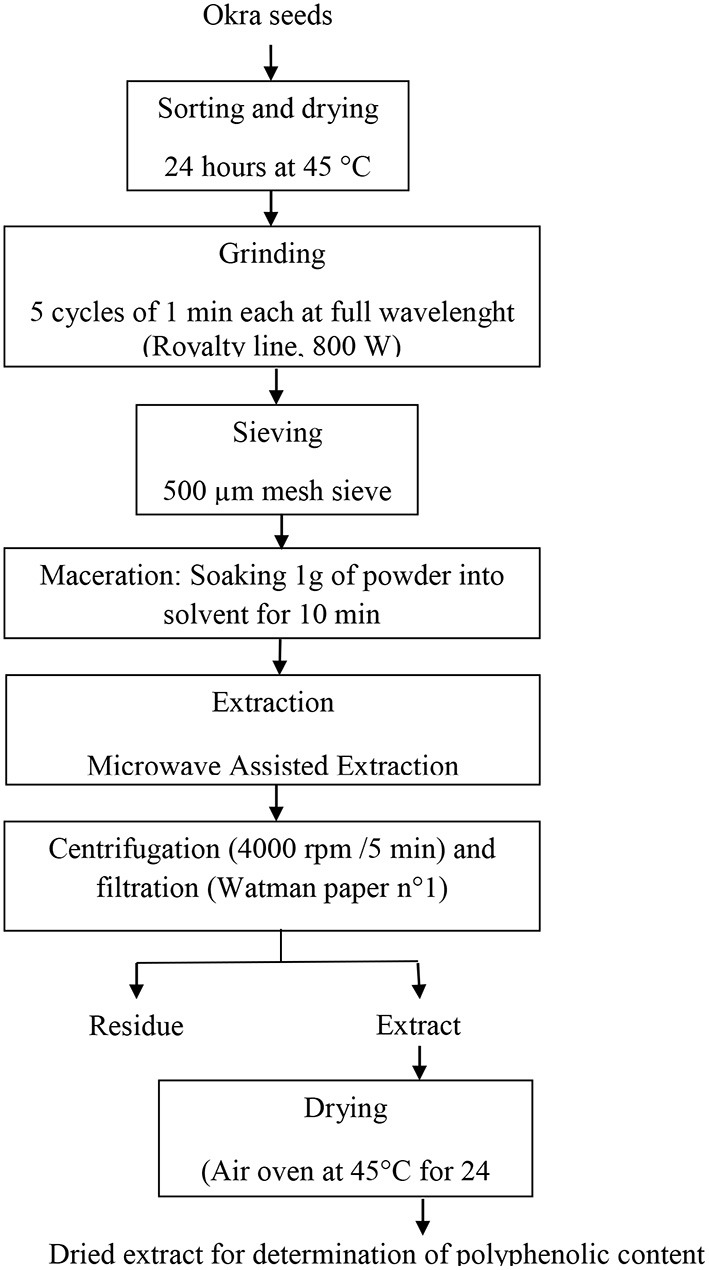
Extraction flowchart of phenols from okra seeds.

For each trial, 1 g of seeds powder was mixed with the appropriate amount of solvent according to the experimental conditions as given by the chosen design. The mixture was stirred using a magnetic agitator, afterward, it was allowed to rest for 10 min at room temperature and put in a microwave oven (SAMSUNG M735) for extraction, under specified conditions. Samples were centrifuged (4000 rpm /5 min) and the supernatant was collected after filtration through Whatman paper n°1. Solvent was then evaporated in an air oven at 45°C until obtention of the dry extract. Dry extracts were immediately used for determination of polyphenolic content. Figure 1 depicts the global flowchart of the work.

##### Determination of total phenolic content

The total phenolic content was assessed according to the protocol described by (15). Briefly, 0.2 mL of Folin reagent (ten-fold diluted) was added to a tube containing 0.01 mL of plant extract (5 mg/mL) and 1.39 mL of distilled water. The mixture was allowed to stand for 3 min before addition of 0.4 mL of sodium carbonate (20% w/v), and then mixed using a vortex. The tube was then incubated at 40°C for 20 min in a water bath and absorbance was read at 760 nm against a blank using a BIOMATE spectrophotometer. Gallic acid (0.2 g/l) was used to draw a calibration curve. All experiments were carried out in triplicates and results were expressed as mg of gallic acid equivalent (GAE) per g of dry extract (mg GAE/g dry weight).

### Optimization of the responses using the central composite design

A face Centered composite design was used to optimize the response, total phenolic content (Y_1_). Ranges of different factors were taken based on literature (6). Experiments were randomized and responses evaluated in triplicates. The proposed model was:


$$\begin{array}{l} \text{Y}\;=\;{\text{a}}_{0}+{\text{a}}_{1}{\text{X}}_{1}+{\text{a}}_{2}{\text{X}}_{2}+{\text{a}}_{3}{\text{X}}_{3}+{\text{a}}_{11}{\text{X}}_{1}{}^{2}+{\text{a}}_{22}{\text{X}}_{2}{}^{2}+{\text{a}}_{33}{\text{X}}_{3}{}^{2}\\ \;\;\;+{\text{a}}_{12}{\text{X}}_{1}{\text{X}}_{2}+{\text{a}}_{13}{\text{X}}_{1}{\text{X}}_{3}+{\text{a}}_{23}{\text{X}}_{2}{\text{X}}_{3} \end{array}$$

Where Y is the response (phenolic content), X_1_, X_2_, X_3_ are the studied factors, a_0_ is the offset term while a_1_, a_2_, a_3_ are linear effects, a_11_, a_22_, a_33_ the quadratic effects and a_12_, a_13_, a_14_, a_23_, a_34_ are interaction effects. Table 1 shows the experimental design in coded and real variables.

**Table 1:** Experimental design in coded and real variables.

**Trial**	**Matrix of real and coded variables**		
	**Time (min)**	**Solvent (mL/g)**	**Power (W)**
1	4.00 (−1)	30.00 (−1)	180.00 (−1)
2	15.00 (+1)	30.00 (−1)	180.00 (−1)
3	4.00 (−1)	80.00 (+1)	180.00 (−1)
4	15.00 (+1)	80.00 (+1)	180.00 (−1)
5	4.00 (−1)	30.00 (−1)	480.00 (+1)
6	15.00 (+1)	30.00 (−1)	480.00 (+1)
7	4.00 (−1)	80.00 (+1)	480.00 (+1)
8	15.00 (+1)	80.00 (+1)	480.00 (+1)
9	0.25 (−1.68)	55.00(0)	330.00(0)
10	18.74 (+1.68)	55.00(0)	330.00(0)
11	9.50(0)	12.95 (−1.68)	330.00(0)
12	9.50(0)	97.04 (+1.68)	330.00(0)
13	9.50(0)	55.00(0)	77.73 (−1.68)
14	9.50(0)	55.00(0)	582.26 (+1.68)
15	**9.50(0)**	**55.00(0)**	**330.00(0)**
16	**9.50(0)**	**55.00(0)**	**330.00(0)**
17	**9.50(0)**	**55.00(0)**	**330.00(0)**
18	**9.50(0)**	**55.00(0)**	**330.00(0)**
19	**9.50(0)**	**55.00(0)**	**330.00(0)**
20	**9.50(0)**	**55.00(0)**	**330.00(0)**

Bold values are replicates of the center points.

### Characterization of the extract

#### Determination of the total flavonoid content

The Total Flavonoid Content (TFC) was obtained using the method described by (16). Sodium nitrite of 0.03 mL (5%) was added to a tube containing 1.49 mL of water and 0.1 mL of extract solution (5 g/mL). After 5 min, a volume (0.003 mL) of aluminum chloride (10%) was added to the tube and the mixture was allowed to rest for 6 min. Afterward, 0.3 mL of NaOH (1M), and 0.24 mL of distilled water were introduced into the tube and mixed with a vortex before absorbance was read at 510 nm against a blank. The calibration curved was made using catechin. All experiments were made in triplicates, and results were expressed as mg of catechin equivalent per g of dry extract (mg CE/g of dry weight).

#### Determination of crude fiber content

Crude fibers were quantified using the *Ceramic Fiber Filter* method as described by (17). These extracts and powders were previously treated to remove lipids using hexane (24 h soaking of 6 g of extracts and powders in 30 mL of hexane with gentle stirring). Briefly, 100 mL of 1.25% H_2_SO_4_ was added to 1 g of lipid-free powder in a round bottom flask and the mixture boiled under reflux for 30 min. The hot solution was quickly filtered under suction and the insoluble matter washed several times with hot distilled water until it was acid free. It was quantitatively transferred into the flask and 100 mL of hot 1.25% sodium hydroxide (NaOH) solution was added and the mixture boiled again under reflux for 30 min before it was quickly filtered under suction. The soluble residue was washed with boiling water until it was base free. Afterwards, it was dried to constant weight in the oven at 105°C, cooled in a desiccator and weighed. The weighed sample (C1) was incinerated in a muffle furnace at 300^o^C for about 2 h, cooled in the desiccator and weight measurement repeated (C2). The loss in weight of sample on incineration was given by C1–C2 while the crude fiber content was expressed as follows:


$$\begin{array}{l} \text{\% Crude fiber}=\frac{C1-C2}{Weight\;of\;original\;sample}\times 100 \end{array}$$

#### Saponin content

The saponin content was estimated as previously described by Koziol (18). Briefly, 0.5 g of the formulation was introduced in a graduated test tube, and 5 mL of distilled water was added.

The tube was closed and vigourously shaken for 30s, and the foam height was immediately measured. The saponin content is linked to the foam height by the following formula:


$$Saponin(mg)=\frac{\left[(\text{0,432})(\text{Foam height after 5 -10 s})+\text{ 0,008}\right]}{\text{ Sample weight (g})}$$

#### Zinc content

Zinc content was determined using the protocol established by Pauwels et al. (19). The sample (1 g) was carbonized at 450 °C for 2 h in an oven (Carbolite Eurotherm), then digested with 10 mL of nitric acid 1N during 30 min. after cooling down, the solution was filtered using Whatman N°1 filter paper in a 50 mL flat bottom flask. Distilled water is added to the filtrate to bring the final volume up to 50 mL. Filtrate (20 mL) + NH_4_Cl (20 mL) + concentrated HCl (1 mL) + sodium sulfite (1 drop) + potassium ferrocyanate (1 mL; 0.5%) were mixed and the mixture allowed to rest for 5 min in dark before absorbance were read using UV-visible spectrophotometer at 650 nm.

### Identification of chemical functions and the number of bioactive compounds

Fourier Transformed Infra-Red Spectroscopy (FTIR) was used to identify the main chemical groups present in the extract while TLC was used to estimates the number of bioactive compounds or groups of compounds present in the extract to identify some.

#### Fourier Transformed Infra-Red Spectroscopy

Spectra were collected at 4000–400 cm^−1^ using a FT-IR Spectrometer (Alpha, Bruker, Germany) on a diamond plate at 4 cm^−1^. Two replicates spectra of 50 scans each were recorded. Raw spectra were corrected.

#### Thin layer chromatography (TLC)

TLC was performed using a pre-coated plate with 60F250 silica gel (MERCK). Two standards were used: catechin and quercetin (1 mg dissolved in 50 ml of ethanol, centrifuged, and the supernatant used). Development was done for 20 min in a pre-saturated (30 min) rectangular development chamber. The mobile phase was made of ethyl acetate/formic acid/ glacial acetic acid/water. The plate was dried at 45 °C in air oven and visualized under UV light (254 nm). Bands were circled and Rf calculated.

### Antioxidant activities

#### Ferric reducing ability of plasma (FRAP)

Ability of extracts and formulation to reduce ferric iron was tested as described by Oyaizu (20).

Briefly, 75 μL of extract/powder suspension was added to 2 mL of FRAP reagent **(**300 mM acetate buffer: pH 3.6; 10 mM TPTZ [(2, 4, 6-tris (2-pyridyl)-S-triazine)**]** in 400 mM of HCl; 10 mM ferric chloride). After 30 min of incubation at room temperature, the absorbance was read at 593 nm against a blank. BHT was used as standard.

#### DPPH inhibitory activity

DPPH inhibitory activity of the extract was assessed using a previously described method (21). Briefly, 100 mL of ethanol was introduced in tubes a_2_-d_2_, a_3_-d_3_, a_4_-d_4_, a_5_-d_5_, and a_6_-d_6_. Afterward, the extract was added in tubes a_1_-d_1_ and a_2_-d_2_. Dilutions were made starting from tubes a_2_-d_2_ to have final concentrations of 200; 100; 50; 25; 12,5 et 6,25 μg/mL. Finally, 900 μL of DPPH solution was added in tubes b_1_-b_6_, c_1_-c_6_ et d_1_-d_6_ while the same volume of ethanol was added in tubes a_1_-a_6_. The mixtures were allowed to stand for 30 min in dark before absorbance were read at 517 nm against blanks prepared in the same conditions (a_1_-d_1_).

**Figure:**
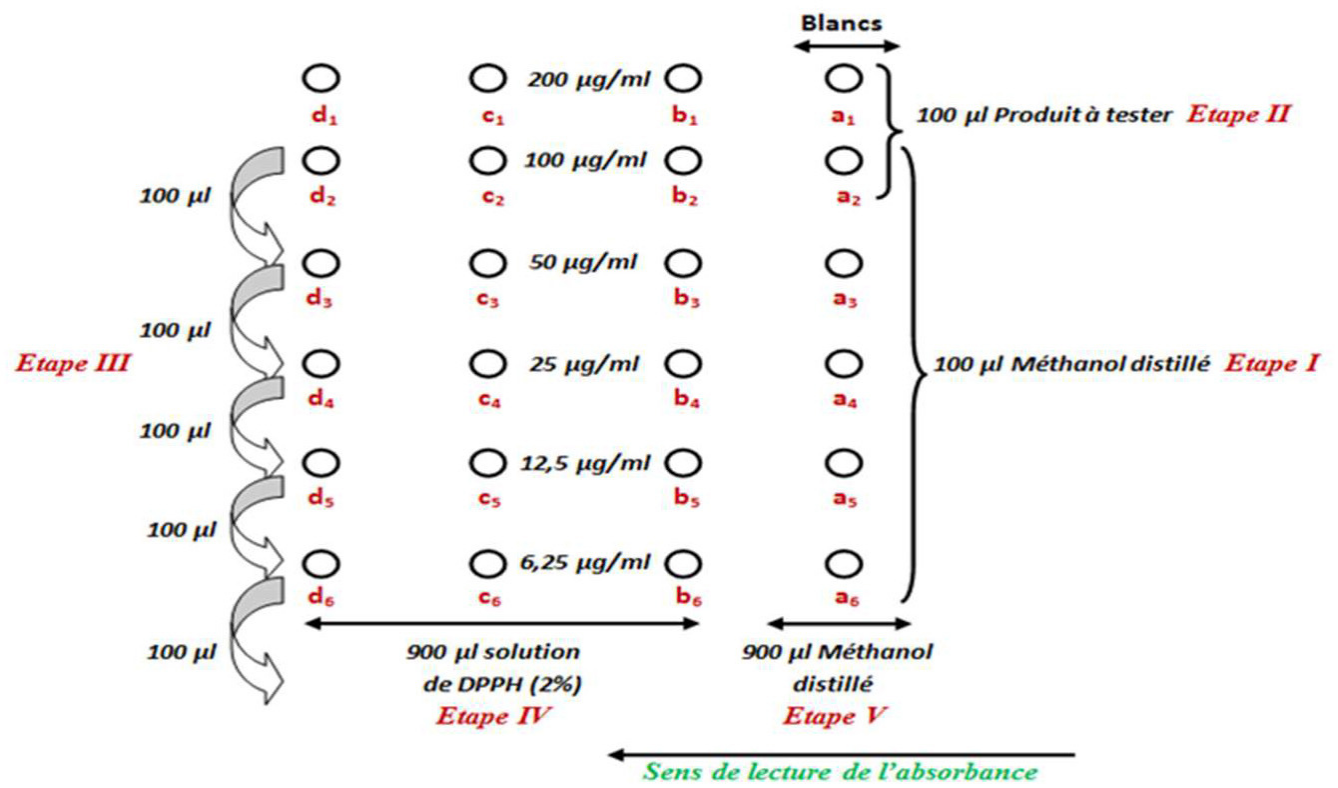


DPPH inhibitory activities (%) were calculated using the formula:


$$\begin{array}{l} \%=\frac{\text{DOc}-\text{DOt}}{\text{DOc}}\text{ x }100 \end{array}$$

Where Doc = absorbance of the control; Dot = absorbance of the test.

Values of IC_50_ (concentration of product inhibiting 50% of DPPH°) were determined using percentages of antioxidant activities and were expressed in μg/mL.

### Alpha amylase inhibitory activity and mechanism of action

The alpha amylase inhibitory activity was determined as described by (22) with slight modifications. Decimal dilutions of the extract were made ranging from 12.5 μg/mL to 200 μg/mL. To 20 μL of extract, 20 μL of porcine pancreatic amylase (0.5 mg/mL) prepared in phosphate buffer (0.02 M, pH 6.9) was added. The mixture was pre-incubated at 25 °C for 10 min before introduction of 20 μL of freshly prepared starch (1% w/v in distilled water). Tubes were incubated at 25° C for 10 min, afterward, 40 μL of DNS was added and the tubes boiled for 15 min to stop the reaction and quantify reducing sugars. 600 μL of distilled water was added to the tubes before absorbance were read at 540 nm. The control was made up of the same constituents with the extract replaced by the buffer.

Percentages of inhibition of each concentration were calculated as follow:


$$\begin{array}{l} \text{\% inhibition}=\frac{DOc-DOt}{DOc}*100 \end{array}$$

Where D0c = absorbance of control and DOt = absorbance of the test.

The mechanism of alpha amylase inhibition was assessed using the same procedure as described before. The formulation was used at a concentration of 100 μg/mL with different concentrations of substrate (1.25, 2.5, 5 and 10 mg/mL). 1/V = f (1/S) (where V = velocity and S = substrate concentration) graph was plotted for determination of the mode of inhibition.

### Acute toxicity

The acute toxicity of the extract was evaluated as per recommendations of OCDE, on evaluation of the acute toxicity of chemical products (23). Two groups of six female rats each, aged 8-12 weeks and weighing between 140 and 180 g, fed on normal chow and received tap water *ad libitum*. The treated group received a single dose of the okra seed extract (5000 mg /Kg of body weight) by oral gavage, while the control received a vehicle at the same dose. Animals were attentively observed for 2 h following administration of the extract, and after each six (06) h during the first day before a daily observation for14 days. Animals were sacrificed and theirs organs collected, observed, weighed and compared to those of the control.

### Oral glucose tolerance test (OGTT)

After an overnight fasting (8 h), animals were given water or the extract at 200 mg / kg of body weight before receiving (5 min after) a D–glucose solution (2 g / kg of body weight). The blood glucose(expressed in mg/dL) was then measured(5–10 μL from tail tip)after 0 min (T_0_), 15 min (T_1_), 30 min (T_2_), 60 min (T_3_), 90 min (T_4_) and 120 min (T_5_) using a portable glucometer (Accu-Chek).

### Antidiabetic effect in high fat high sucrose + streptozotocin induced diabetes

#### Induction of diabetes

Human type 2 close diabetes mellitus was induced on obese albinos Wistar rats by single intra peritoneal administration of 40 mg/Kg body weight of a freshly prepared streptozotocin solution. Streptozotocin was prepared in citrate buffer (0.1 M, pH 4.5). After streptozotocin administration, blood obtained from tail puncture was used to assess fasting blood glucose (FBG) of the animals 3 days later and those with a FBG≥ 1.26 dg/dL were considered diabetic and used for the experiments.

Rats were divided into 4 groups of six rats each as follow:

T-: Normal control (healthy rats).
T+: Diabetic control (untreated obese and diabetic rats).
Okra: Obese and diabetic rats treated with okra seeds' extract at 250 mg/Kg of body weight once per day.
Met: Obese and diabetic rats treated with metformin at 0.25 mg/Kg body weight once per day.

#### Fasting blood glucose, food intake and body weight

Blood was collected by cardiac puncture for estimation of plasma biomarkers, while samples of key organs like liver, kidneys, heart, pancreas and lungs were collected for evaluation of specific markers (ALT, AST and ALP) and estimated oxidative stress markers at their levels.

#### Blood lipid profile and atherogenic index

Serum Triglyceride (TG), Total cholesterol (T Chol) and HDL cholesterol (HDL Chol) was determined according to the procedure describe on the commercial kit used (MONLAB). LDL cholesterol was estimated using the equation of Friedewald et al. (24):


$$\begin{array}{l} \text{LDL = }total\;Cholesterol-[HDL\;Cholesterol\\ \text{ +}(Triglycerides/n)] \end{array}$$

*n* = 2, if results are expressed in mmol/L and *n* = 5, when results are expressed in mg/dl. The atherogenic index was calculated as follow:


$$\text{Atherogenic index=}\frac{\text{ Total cholesterol }}{\text{HDL cholesterol }}$$

#### Renal, hepatotoxicity and oxidative stress markers

Renal and hepatoprotective activity of the okra seeds extract was assessed by evaluating key biomarkers. Alanine Amino-Transferase (ALT), Aspartate Amino-Transferase (AST) and Alcaline Phosphatase (ALP) were tested to investigate liver functions, while plasmatic and urinary creatine levels were studied to monitor kidneys functions. All measurements were made as described in the commercial kits used (Teco Diagnostics, USA).

Malondialdehyde (MDA) level and reduced glutathione (GSH) were evaluated as oxidative stress markers in the plasma and at the level of the key organs up mentioned. MDA was determined according to the method described by Yagi (25) while GSH was determined as per the method of Ellman (26).

## Statistical analysis

Designing and analysis of the results were done using Minitab 18. Experiments were carried out in triplicates. Statistical significance of the variables was determined at 5% probability level. Main effects and contour plots were plotted using Sigma Plot v11.0 (c) Systat. Data on phenol and flavonoid contents, as well as those on biochemical parameters were expressed as mean± SD and analyzed by One way Analysis of variance (ANOVA) using SPSS version 22 (IBM). Comparison were made using Bonferroni test at 5% significance.

## Results and discussion

### Optimization of the extraction of polyphenols from okra seeds using the central composite design

Three factors were studied for extraction of polyphenols from okra seeds, namely the microwave wavelength, the time of extraction and the solvent/dry matter ratio. Ethanol proportion was decided to be 50% based on previous work. Table 2 represents the experimental and predicted response values in different variable conditions given in real and coded values.

**Table 2:** Experimental and predicted responses.

**Trials**	**Matrix of real and**			**Responses**	
	**coded variables**				
				**TPC (mg GAE/g)**	
	**Time (min)**	**Solvent (mL/g)**	**Power (W)**	**Exp**	**Pre**
1	4.00 (−1)	30.00 (−1)	180.00 (−1)	57.27 ± 1.09	51.97
2	15.00 (+1)	30.00 (−1)	180.00 (−1)	55.27 ± 2.02	52.77
3	4.00 (−1)	80.00 (+1)	180.00 (−1)	71.31 ± 2.00	69.05
4	15.00 (+1)	80.00 (+1)	180.00 (−1)	64.65 ± 1.60	67.76
5	4.00 (−1)	30.00 (−1)	480.00 (+1)	56.69 ± 3.33	52.95
6	15.00 (+1)	30.00 (−1)	480.00 (+1)	47.65 ± 2.21	49.28
7	4.00 (−1)	80.00 (+1)	480.00 (+1)	58.84 ± 1.09	60.71
8	15.00 (+1)	80.00 (+1)	480.00 (+1)	50.28 ± 1.13	54.95
9	0.25 (−1.68)	55.00 (0)	330.00 (0)	45.45 ± 2.33	50.75
10	18.74 (+1.68)	55.00 (0)	330.00 (0)	51.00 ± 1.59	46.58
11	9.50 (0)	12.95 (−1.68)	330.00 (0)	58.24 ± 0.90	63.83
12	9.50 (0)	97.04 (+1.68)	330.00 (0)	87.66 ± 3.33	82.96
13	9.50 (0)	55.00 (0)	77.73 (−1.68)	53.22 ± 1.09	57.04
14	9.50 (0)	55.00 (0)	582.26 (+1.68)	50.03 ± 3.45	47.09
15	**9.50 (0)**	**55.00 (0)**	**330.00 (0)**	68.36 ± 1.30	68.12
16	**9.50 (0)**	**55.00 (0)**	**330.00 (0)**	68.24 ± 1.90	68.12
17	**9.50 (0)**	**55.00 (0)**	**330.00 (0)**	67.89 ± 2.00	68.12
18	**9.50 (0)**	**55.00 (0)**	**330.00 (0)**	68.54 ± 3.10	68.12
19	**9.50 (0)**	**55.00 (0)**	**330.00 (0)**	68.01 ± 2.89	68.12
20	**9.50 (0)**	**55.00 (0)**	**330.00 (0)**	67.84 ± 1.09	68.12

Exp, experimental; Pre, predicted; Bold values are replicates of the center points.

### Analysis of main effects

The entire experimental plan consisted of 20 trials. The highest polyphenolic content (87.66 ± 3.33 mg of GAE/g) was obtained at 330 W for 9.5 min of treatment time with 97.04 mL of solvent. The lowest content (45.45 ± 2.33 mg GAE/g) was observed at 330 W of microwave power with 55.00 mL of extracting solvent and a heating time of 0.25 min. These values are greater than those of Peter et al. (27) and Hu et al. (28) who obtained a total phenolic content of 20.2 and 21.1 mg GAE/g from okra seeds by water and methanol extraction respectively. The highest phenolic content was also greater than what obtained by Geng et al. (11) from okra flowers by ethanol extraction (40.77 ± 0.83 mg GAE /g material). Such differences could be related to the extraction method. Microwaves induce a quick elevation of the temperature, thus leading to a rapid breakdown of cell walls and liberation of polyphenols out of the matrix (5).

### Effect of time

**Figure 2:**
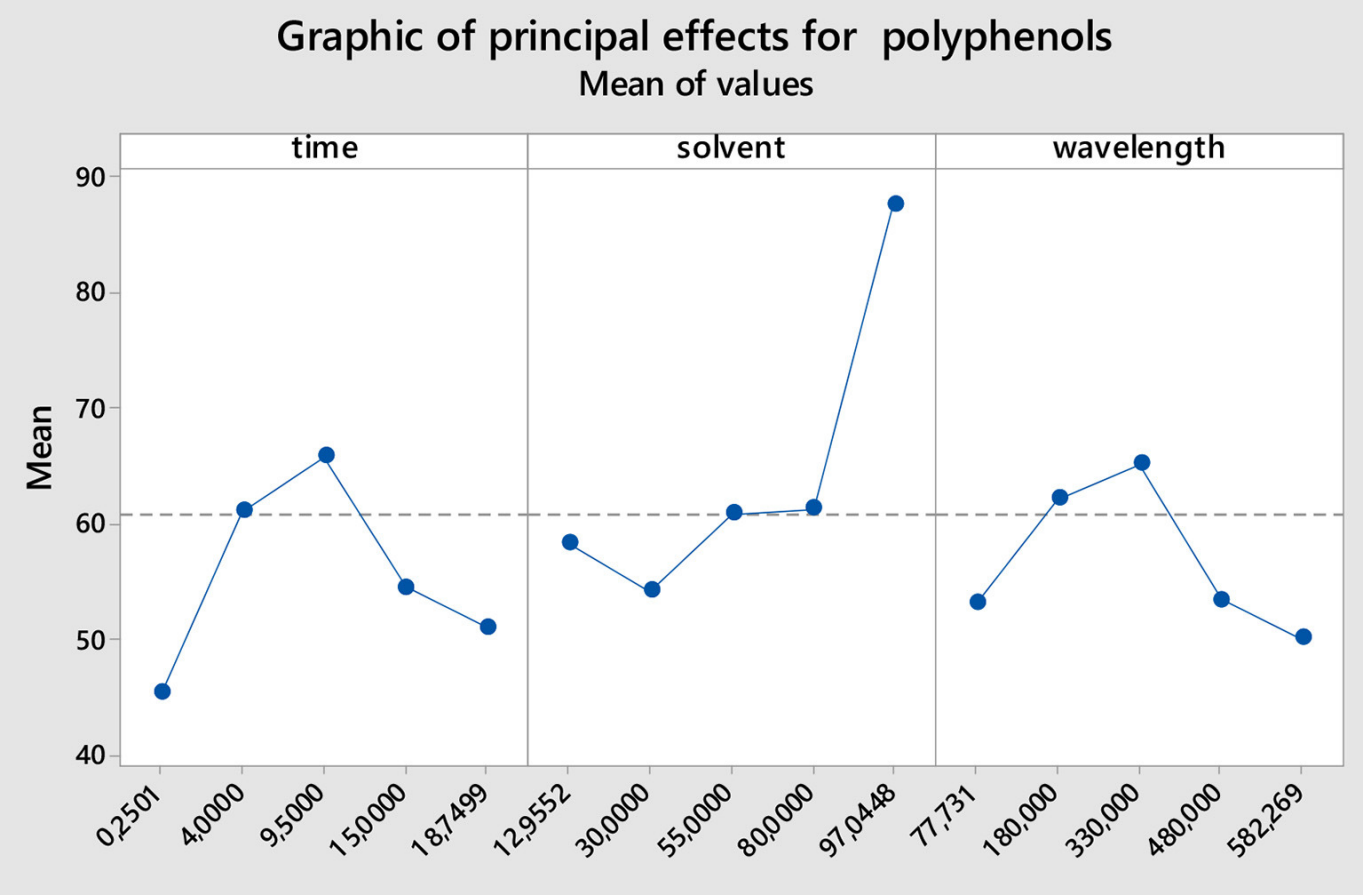
Main effect plots of individual factors on polyphenolic content.

The effect of time on the total phenolic content is illustrated in Figure 2. Increase in the exposition time from 2 to 9.5 min led to an increase in the phenolic content, probably due to the breakdown of cell walls under the heat generated by the microwave, thus leading to a progressive liberation of polyphenols in the solvent system. From 9.5 min, any increase in the extracting time leads to a progressive reduction in the polyphenolic content of the extract obtained. This may be the result of progressive destruction of these thermo-sensitive compounds under long exposure to heat. Previously, the similar effect has also been noticed by Sanja et al. (29) and Xuan et al. (30).

### Effect of solvent ratio

An almost linear increase in the phenolic content of the extracts was observed for any increase in the solvent/dry matter ratio (Figure 2). The highest content was obtained with the ratio 97.04 mL/g thus suggesting that high solvent ratio increases mobility of compounds (mass transfer) from plant matrix to the solvent system, as already reported by (31).

### Effect of wavelength

It can be seen from Figure 2 that any increase in the extracting power from 77.73 to 330 W induced an increased in the polyphenolic content of the okra seed extracts as a result of more break down of cell walls under the increased heat in the operating system alongside with the power increase, which led to more liberation of polyphenols in the solvent. But any increased in the power of the microwave apparatus above 330 W caused a progressive diminution of the polyphenolic content of the extracts as a consequence of degradation of these compounds exposed to high temperature, since usage of high power in microwave apparatus induce a quick and high elevation of the solvent temperature even when exposure is for a short duration (29).

#### ANOVA, regression equations for the responses

Table 3 below shows the ANOVA and the influence of each independent factor. Taken individually, solvent/dry matter ratio and the power of the microwave apparatus significantly influenced (*p* < 0.05) the polyphenolic content of the extracts. The quadratic effect of the time (X_1_X_1_) and power of extraction (X_3_X_3_) significantly influenced the total phenolic content of the extracts, with the greatest contribution in the response (30.86%) for the time, followed by the quadratic effect of the microwave power (22.87%). The mathematical model predicting the effect of the factors on the response is given below (Eq. 1):


$$\begin{array}{l} \text{TPC}\;=\;7.8\;+\;4.75\;{\text{X}}_{1}+\;0.141\;{\text{X}}_{2}\;+\;0.1938\;{\text{X}}_{3}\;-\;0.2274\;{\text{X}}_{1}{\text{X}}_{1}\\ \;+\;0.00298\;{\text{X}}_{3}{\text{X}}_{3}\;-\;0.000252\;{\text{X}}_{2}{\text{X}}_{2}\;-\;0.0038\;{\text{X}}_{1}{\text{X}}_{2}\\ \;-\;0.00135\;{\text{X}}_{1}{\text{X}}_{3}\;-\;0.000621\;{\text{X}}_{2}{\text{X}}_{3} \end{array}$$

TPC: total phenolic content; X_1_: time; X_2_: power; X_3_: solvent/dry matter ratio.

**Table 3:** Evaluation of quadratic model: *P*-value, F value, RC, CF (Contribution Factor) (%), AADM and Bf for phenols.

**Source**	***p*-value**	***F* value**	**CF (%)**
Time (X_1_)	0.347	0.98	1.04
Solvent (X_2_)	**0.001**	20.50	21.77
Power (X_3_)	**0.040**	5.55	5.89
X${}_{1}^{*}$ X_1_	**0.000**	31.63	30.86
X${}_{2}^{*}$X_2_	0.158	2.32	4.22
X${}_{3}^{*}$ X_3_	**0.001**	21.53	22.87
X${}_{1}^{*}$ X_2_	0.757	0.10	0.11
X${}_{1}^{*}$ X_3_	0.512	0.46	0.49
X${}_{2}^{*}$ X_3_	0.186	2.01	2.14
**Validation of the model**			
R^2^	0.89		
AADM	0.00		
Bf	1.00		

Bold, Factors that significantly (p < 0.05) influenced the polyphenolic content.

#### Assessment of model quality and optimum conditions

Experimental values showed that these mathematical models can well explain the observed results. According to (32), a good mathematical model should predict at least 75% of the responses; R^2^ should then range 0.75 to 1. Based on the determination coefficient for phenols (0.89) given in Table 3, it was concluded that the postulated second-order polynomial equations, truly represented the experimental data. Also, obtaining values of AADM (Analysis of the Absolute Average Deviation) and Bf (Bial factor) respectively equal to 0 and 1 confirmed the suitability of the models since values were in the normal range (0 for AADM and 0.75<Bf<1.25 for Bf).

#### Optimization of the process

**Figure 3:**
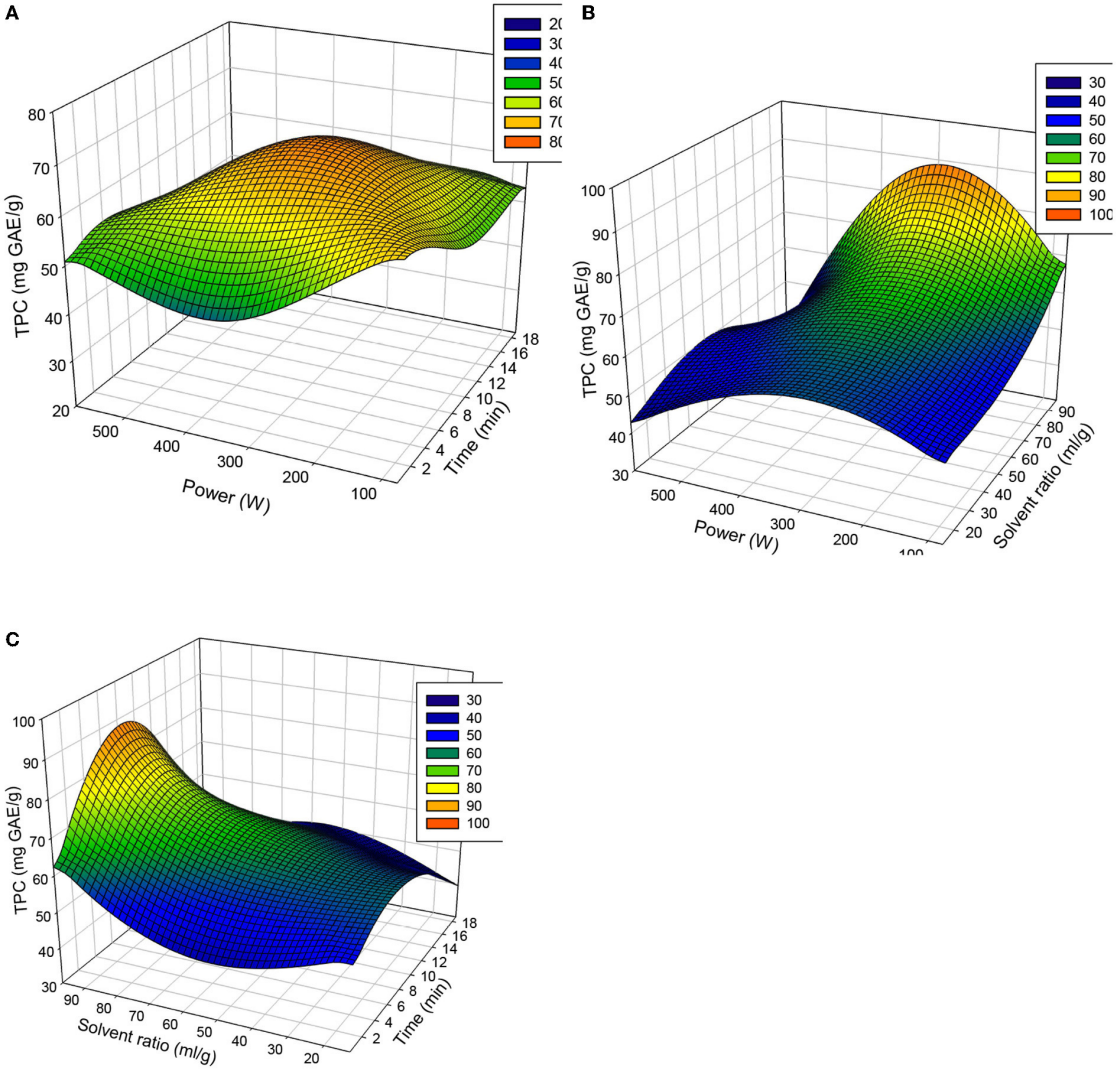
**(A–C)** Response-surface curves for phenolic content considering the different factors taken 2 by 2.

After validation of the model, the optimal extraction conditions for extracting phenols from okra seeds were determined using response surface curves. Figures 3A–C illustrates the variation in the polyphenolic content of okra seed extracts under the influence of different factors taken two by two, drawn with Minitab 18. These figures show that maximum content of phenolic content (87.66 ± 3.33 mg of GAE/g) is obtained at 330 W, with a solvent ratio of 97.04/1 for 9.5 min.

#### Confirmation experiments

Replications of the optimum conditions proposed by the model were made in order to confirm the quality of our model to predict the optimal conditions for TPC. No significant difference was noticed between optimal predicted and experimental values obtained, thus confirming the validity of the predicted optimal value given by the software as shown in Table 4. The optimization process permitted to determine the conditions of maximum extraction of polyphenols from okra seeds' as a TPC of 86.77 ± 1.52 mg GAE/g were obtained, a value two-fold greater than the maximum content of 39.39 ± 7.46 mg GAE/g obtained by Graham et al. (33) on different okra seeds cultivars.

**Table 4:** Experimental, predicted values and desirability for polyphenolic content in optimal conditions.

	**Predicted value**	**Experimental value**	**Desirability**
Phenol (mg GAE/g)	85.12^a^	86.77 ± 1.52^a^	0.93

On the same line, values with different letters significantly differ (*p* > 0.05).

### Characterization of the extract

#### Phytochemical characterization of the extract

Table 5 show the phytochemical characterization of the extract obtained with the optimal conditions.

**Table 5:** Phytochemical composition of the optimized okra extract.

**Parameters**	**TPC (mg GAE/g)**	**TFC (mg CE/g**	**Fiber (g/100g)**	**Saponin (mg/g)**	**Proteins (mg/g)**	**Zinc (mg/g)**
	86.77 ± 1.52	2.62 ± 0.27	1.84 ± 0.06	0.43 ± 0.05	0.175 ± 0.020	0.003 ± 0.00

TPC, total phenolic content; TFC, total flavonoid content; EC, equivalent catechin.

#### Antioxidant activity

Tables 6, 7 show the antioxidant capacities of the okra extract measured by the FRAP and DPPH scavenging methods. The extract performed better than BHT in both tests. It showed a very high DPPH scavenging ability in a concentration dependent manner with an IC_50_ of 3.99 ± 0.15 μg/mL, at least 7 times smaller than the minimum value obtained by 32. Optimization permitted to produce high antioxidant extract compared to what have been previously reported (28, 33). The great antioxidant capacity of the extract should be related to its high phenolic content, since polyphenols have been reported to possess antioxidant capacities (33).

**Table 6:** Absorbance of the at different concentration during the FRAP assay.

	**Concentration (**μ**g/mL)**				
	**12,5**	**25**	**50**	**100**	**200**
AB	1.51 ± 0.17^b^	3.02 ± 0.06^b^	3.20 ± 0.06^b^	3.15 ± 0.01^b^	3.14 ± 0.03^a^
BHT	0.18 ± 0.02^a^	0.40 ± 0.15^a^	0.45 ± 0.09^a^	0.97 ± 0.01^a^	2.70 ± 0.10^a^

AB, *Abelmoschus esculentus* extract; BHT, Butyl hydroxyl–toluene; ^a, b^in the same column, values with different letters differ significantly (p < 0.05).

**Table 7:** Percentage of DPPH inhibition at different concentrations.

	**Concentration (**μ**g/mL)**					
	**12.5**	**25**	**50**	**100**	**200**	**IC_50_**
AB	50.57 ± 0.18^b^	62.22 ± 0.04^b^	87.18 ± 0.08^b^	91.44 ± 0.01^b^	91.29 ± 0.00^b^	3.99 ± 0.15^a^
BHT	15.84 ± 0.00^a^	20.16 ± 0.09^a^	24.91 ± 0.08^a^	38.63 ± 0.22^a^	53.31 ± 0.15^a^	4.4x10^11^ ± 15.28^b^

AB, *Abelmoschus esculentus* (okra) extract; BHT, Butyl hydroxyl–toluene. ^a, b^in the same column, values with different letters differ significantly (p < 0.05).

#### Thin layer chromatography

**Figure 4:**
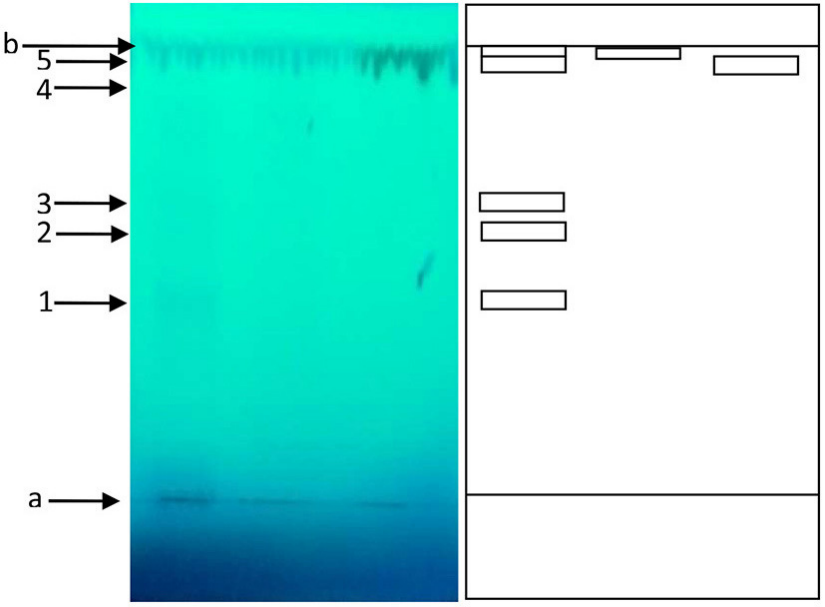
Chromatogram of okra extract under UV 254 light **(left)** and schematic representation **(right)**. a= deposit line, b= front line. 1, 2, 3, 4 and 5 are the different spots observed under UV light (254 nm).

Figure 4 shows the different spots observed on TLC plate. The presence of five (05) spots with Rf ranging from 0.17 to 0.97, led to the conclusion that the optimized extract of okra seeds' contained at least 5 compounds or groups of compounds. Among these compounds, quercetin and catechin were identified with Rf of 0.94 and 0.97 respectively. These observations are in accordance with those reported by Peter et al. (27) and Ong et al. (3) who showed the presence of quercetin and its derivatives in okra seeds' extract. The Rf values of the different spots observed and a tentative identification is given in Table 8.

**Table 8:** Rf and identification of the different spots obtained on TLC with okra seeds' extract.

**Bands**	**1**	**2**	**3**	**4**	**5**
Rf	0.18	0.32	0.59	0.94	0.97
Identification	/	/	/	Quercetin	Catechin

#### FTIR spectral analysis

**Figure 5:**
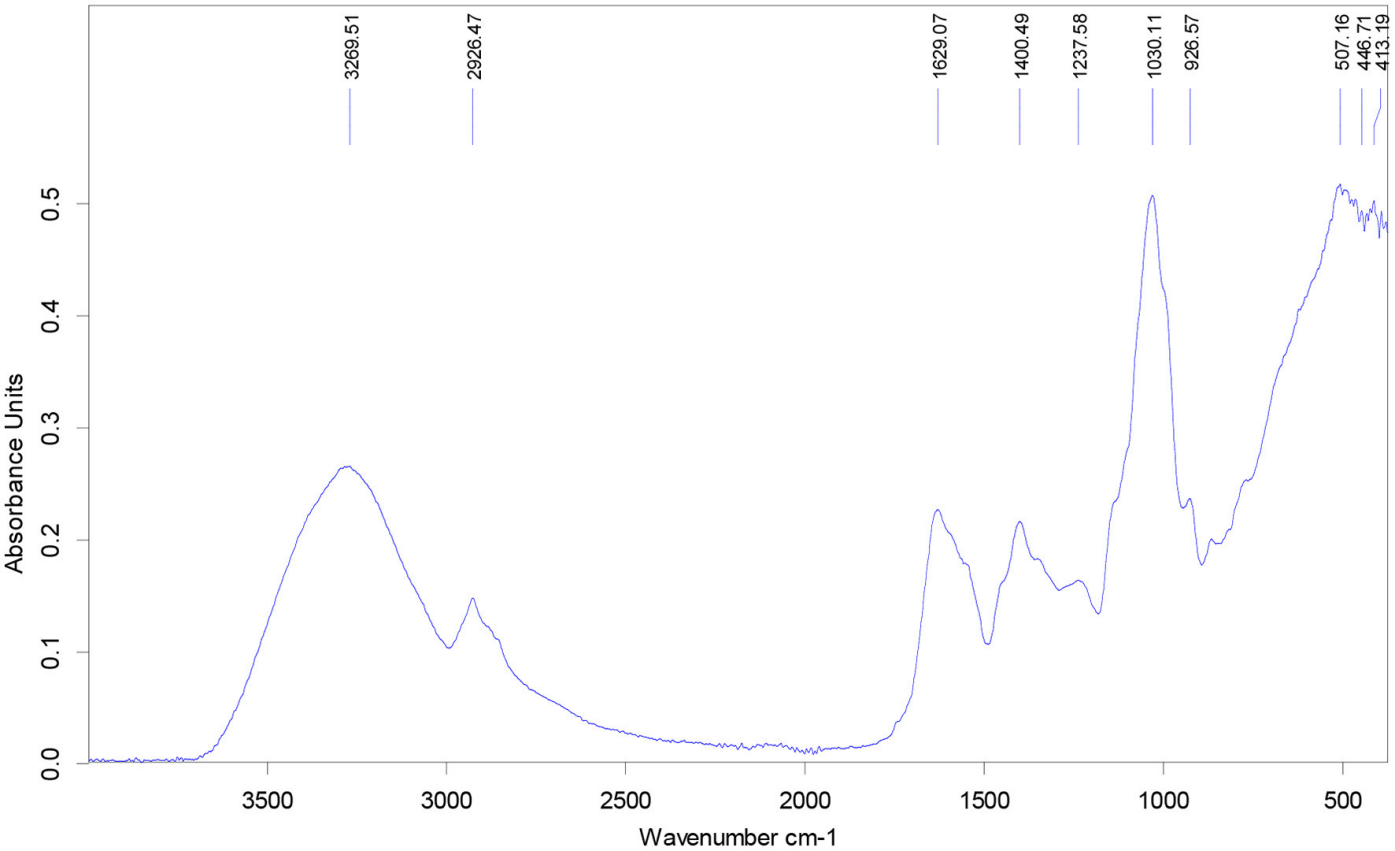
FTIR spectra of the optimized okra seeds' extract.

The functional groups of the bioactive compounds present in the extract were tentatively identified using spectral analysis. Figure 5 is the FTIR spectra of the extract while Table 9 summarizes the different bands obtained and their assignation. Ten (11) bands were observed among which eight were characteristic of molecules possessing antioxidant and antidiabetic activities, including phenols and flavonoids.

**Table 9:** Peak wave numbers and tentative identification.

**Bands (cm^−1^)**	**Vibration**	**Assignation**	**Reference**
3269	–C–OH (stretching)	Water, cabohydrates	(34, 35)
2926	–C–H (stretching)	Aliphatic portion of lipids	(35)
		Isoflavones	(36)
1629	–C=C		(37)
1400	–O–H (bending)	Phenols or tertiary alcohol	(38)
1237	unidentified
1030	Ester –C–O (stretching)	Glycosidic groups	(39)
926	–C–C (stretching)	Alkane: lipids, amino acids, proteins	
507	Phenolic ring (torsion)	Phenols	(40)
446	unidentified
413	–C–OH in plane (bending)	Phenols	(41)

#### Acute toxicity

No visible change on the behavior or the macroscopic aspect of the main organs of the animals, were noted after administration of the extract to normal rats up to 14 days after administration. The optimized extract of okra seeds was then said not to be toxic at unique dosage intake of up to 5000 mg/kg body weight. These results are in accordance with those of (42) who reported that okra hydroalcoholic extract did not show any toxicity or death up to a dose of 5000 mg/kg in Wistar rats. Uddin et al. (43) had also found okra mucilage powder and peel–seed mixture to be safe at a dose level of up to 1000 mg/kg of body weight in mice.

#### Oral glucose tolerance test

Table 10 shows the blood glucose concentrations of animals at different time during the oral glucose tolerance test. The okra seeds' extract limited the increase in the glucose level by 24.20 % compare to the normal control. Also, a quick drop down was noticed in the blood glucose level of rats treated with the optimized okra extract. This could be due the presence of soluble fibers in the extract which adsorbed glucose in the intestine, thus preventing it absorption into the blood (13).

**Table 10:** Blood glucose levels and increment during the OGTT.

	**0 min**	**15 min**	**30 min**	**60 min**	**90 min**	**120 min**	**Increment**
AB	100 ± 7.54^b^	155.33 ±4.5^a^	134 ± 7^a^	124.33 ± 2.08^a^	113.33 ± 4.93^a^	98 ± 8^a^	55.33 ± 3.05^a^
T	85 ± 4.35^a^	158 ± 6.2^a^	141 ± 8.12^a^	126 ± 3.46^a^	116.33 ± 6.65^a^	107.33 ± 7.09^a^	73 ± 2.64^b^

AB, *Abelmoschus esculentus* (okra) extract; T, normal control. Increment: highest blood glucose value (at 15 min)—start value (0min); ^a, b^in the same column, values with different letters differ significantly (p < 0.05).

#### Alpha amylase inhibitory activity and mechanism

**Figure 6:**
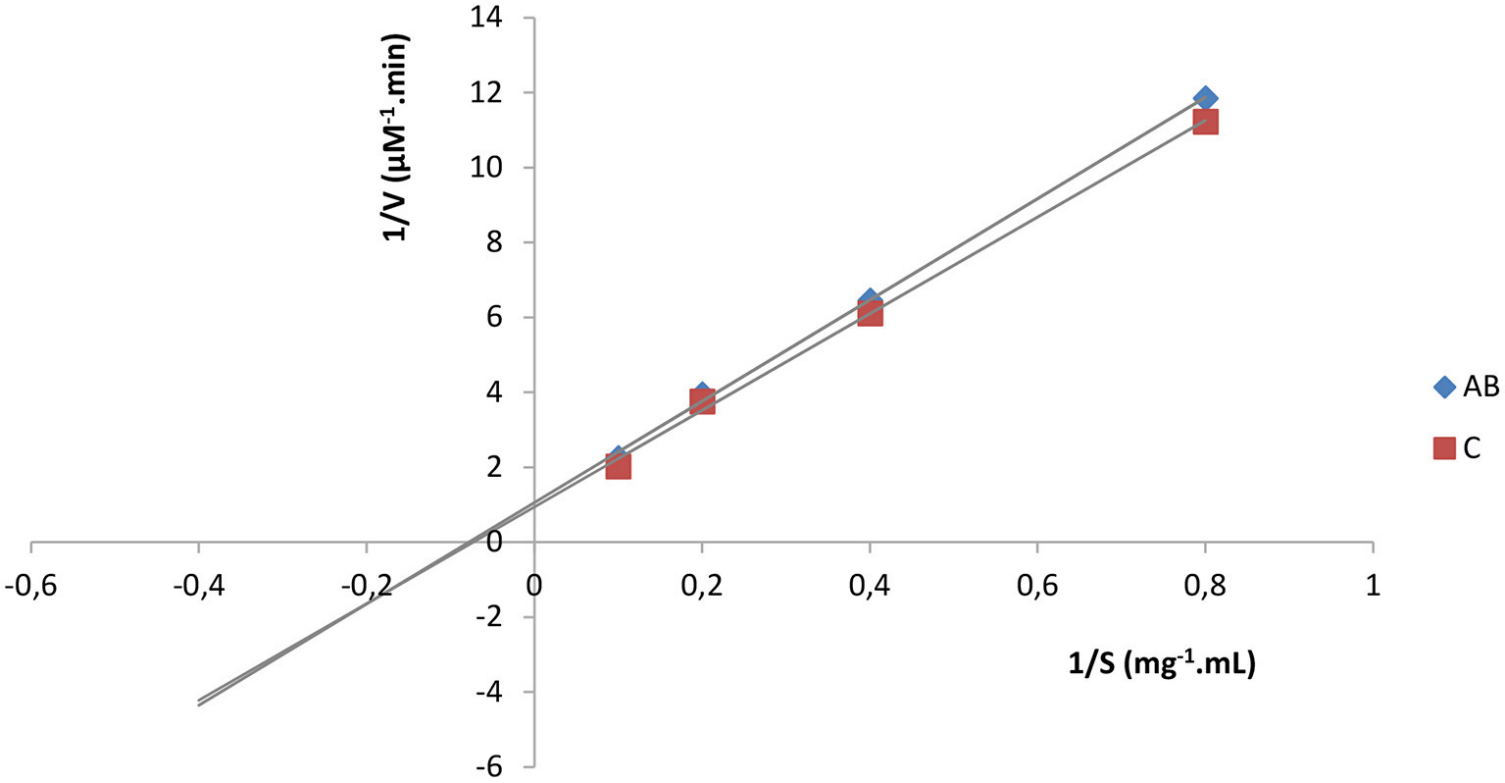
Lineaweaver-Buck plot for AB. AB, *Abelmoschus esculentus* seeds' extract; C, control.

The optimized okra seeds' extract was able to inhibit porcine pancreatic alpha amylase for up to 24.80 % at concentration of 200 μg/mL as shown in Table 11. Lineaweaver-Buck plot permitted to classify the okra extract as a non-competitive inhibitor of alpha amylase (Figure 6). Similar observations were made by Quan et al. (44) who explained that the inhibitory activity could be due to the presence of phenolic compounds in the extract. Also, a possible synergistic interaction between polyphenolic and the terpenoid compounds could justify the great alpha amylase inhibitory activity of certain extracts compared to others (44). The observed result could also be due to the fiber content of the extract since (45) and Nsor-Atindana et al. (46) previously demonstrated that cellulose in a concentration and particles size-dependent manner can inhibit alpha amylase and alpha glucosidase.

**Table 11:** Alpha amylase inhibitory percentages and IC_50_.

	**Concentration (**μ**g/mL)**				**IC_50_ (μg/mL)**
	**25**	**50**	**100**	**200**	
AB	10.04 ± 0.30	10.66 ± 0.76	17.13 ± 2.16	24.80 ± 2.36	484.17 ± 2.33

AB, *Abelmoschus esculentus* (okra) seeds' extract.

### Antidiabetic activity of the optimized okra extract

#### Effect of the treatment on the fasting blood glucose (FBG), the body weight and the food intake

A significant decrease in the FBG was observed in the group treated with the okra extract compared to the negative control. The optimized extract exhibited better hypoglycemic activity than metformin. Such activity could be explained by the presence of polyphenols which are able to stimulate glucose absorption at the muscular level, or to increase insulin production by the pancreas. Flavonoids like quercetin that were found in the okra extract can induce the expression of the glucose transporter GLUT 4 or inhibit PPARγ 1, which end up in an increased absorption of glucose in muscles (47).

At the end of the treatment, a significant weight loss was notice in untreated diabetic rats, as a consequence of diabetes (48). The okra extract was then able to alleviate muscular weight loss in treated diabetic rats, probably by ameliorating the assimilation and utilization of glucose at the levels of cells, the weight loss in diabetics being a direct consequence of the inability of the body to use circulating glucose. Similar observations were made by Gupta et al. (49, 50), with plant extracts on diabetic rats.

A decrease of 2.57 mg in the food intake of the rats treated with the okra extract was observed, thus suggesting the extract had been able to alleviate polyphagia in animals. The observed activity can be related to the soluble fibers contained in the extract, which may limit the production of orexigenic compounds like ghrelin by enhancing the production of appetite suppressors like cholecystikinin, Glucagon-like Peptide 1 (GPL-1) and peptide YY (50–52). Table 12 summarizes the variations in the different parameters.

**Table 12:** Variation of some parameters between the start and the end of the experiment.

	**FBG (g/L)**	**Body weight (g)**	**Food intake (g)**
AB	−102 ±7.81^a^	−2.33 ± 9.01^b^	−2.57 ± 0.04^a^
Met	−83.66 ± 9.23^b^	−7.66 ± 15.94^b^	0.32 ± 0.01^b^
T+	26 ± 2.64^c^	– 45.33 ± 11.06^a^	8.46 ± 0.04^d^
T–	2 ± 1.41^d^	39.5 ± 8.54^c^	4.54 ± 0.02^c^

AB, *Abelmoschus esculentus* (okra) seeds' extract; Met, Metformine; T+, Diabetic control; T–, Negative control; FBG, Fasting blood glucose. Variation = final value–start value; a, b, c, and d, in the same column, values with different letters differ significantly (*p* < 0.05).

#### Effect of the treatment on the lipid profile and the atherogenic index

The treatment with okra extract significantly lowered blood triglyceride (TAG), total cholesterol (T Chol) and LDL cholesterol concentrations in diabetics rats. HDL cholesterol concentration was significantly elevated in the AB group, compared to diabetic and normal controls. The atherogenic risk was significantly reduced in okra treated diabetic controls and metformine treated rats, thus leading to the conclusion that treatment of diabetic patients with okra extract could reduce the risk of cardiovascular accidents. Presence of flavonoids like quercetin (53) or alkaloids (54) in the extract may explain these activities. Esmaeilzadeh et al. (55) reported that okra extracts could stimulate the production of alpha hydroxylase, the enzyme converting cholesterol into bile acids in the liver, or inhibit the HMG-COA (3-hydroxy-3-methyl-glutaryl-coenzyme A) reductase; resulting in a decrease in the circulating cholesterol. The extract could exert its LDL cholesterol lowering activity by limiting the intestinal absorption of lipids implicated in their formation in the liver (56). Also, Brijyog et al. (57) stated that flavonoids present in the extract could induce the transcription of lipoprotein lipase genes. The enzyme is then produced and breakdown lipoproteins like LDL cholesterol with an end result of its blood concentration reduction. Similar observations were made by Abd El Latif et al. (58) who noticed a normalization in the lipid profile of diabetic rats treated with soybean isoflavones. Table 13 gives the lipid profile and atherogenic index of the animals at the end of the treatment.

**Table 13:** Lipid profile and atherogenic index of animals at the end of the treatment.

	**TAG**	**T CHOL**	**HDL**	**LDL**	**AI**
AB	33.41 ± 5.03^a^	47.73 ± 0.23^a^	36.73 ± 1.93^b^	4.32 ± 0.89^a^	1.30 ± 0.06^a^
Met	64.81 ± 1.56^c^	68.40 ± 2.11^b^	36.08 ± 2.60^b^	19.35 ± 3.69^c^	1.90 ± 0.15^b^
T+	42.67 ± 3.61^b^	76.53 ± 4.73^c^	30.94 ± 3.23^a^	37.05 ± 5.83^d^	2.49 ± 0.33^c^
T–	47.43 ± 3.50^b^	64.25 ± 4.59^b^	38.98 ± 4.07^b^	15.78 ± 2.31^b^	1.65 ± 0.08^b^

AB, *Abelmoschus esculentus*; F, Formulation; Met, Metformin; T+, Diabetic control T–, Normal control. TAG, Triglycerides; T CHOL, Total cholesterol; HDL, HDL cholesterol; LDL, LDL cholesterol; AI, Atherogenic index; a, b, c, and d, in the same column; values with different letters differ significantly (*p* < 0.05).

#### Renal and hepato-protective activities of the optimized okra extract

Serum Aspartate amino transferase (AST), Alanine amino transferase (ALT) and Alcaline Phosphatase (ALP) concentrations were measured to assess the integrity of the liver. The liver plays a key role in the glucose homeostasis, and diabetes could disturb its functioning, which is marked by elevated concentrations of AST, ALT and ALP in the blood, resulting from an inflammatory state induced by hyperglycemia and oxidative stress (59, 60). The okra extract exhibited hepato-protective capacities by significantly reducing these enzymes concentrations in the blood, compared to diabetic control. Similar observations were made by (58, 61, 62). The hepato-protective activity noticed with the optimized okra extract could be due to its antioxidant capacity, as (63) hypothesized that plant extracts could exert their hepato protective activity by combating the oxidative stress which is responsible of the inflammation and necrosis of the liver, resulting in the elevation of the above mentioned liver enzymes and precisely the ALP. Table 14 summarizes the values obtained.

**Table 14:** Effect of the treatment on liver biomarkers.

	**AST (UI/l)**	**ALT (UI/l)**	**ALP (UI/l)**
AB	38.11 ± 6.71^a^	8.75 ± 1.54^a^	214.65 ± 5.18^b^
Met	74.08 ± 5.92^b^	21.19 ± 3.75^b^	209.15 ± 4.85^b^
T+	89.83 ± 5.05^c^	28.58 ± 4.04^c^	231.17 ± 2.59^c^
T–	40.83 ± 6.06^a^	22.45 ± 3.94^b^	137.60 ± 7.78^a^

AB, *Abelmoschus esculentus*; Met, Metformine; T+, Diabetic control; T–, Normal control; AST, Aspartate amino transferase; ALT, Alanine amino transferase; ALP, Alcaline Phosphatase; a, b, and c, in the same column; values with different letters differ significantly (*p* < 0.05).

Among the most severe complications of diabetes, is renal failure. A good antidiabetic management should then prevent or delay it appearance. Creatinin clearance is the commonest way to assess the renal status since a high concentration of creatinin in the blood circulation can be indicative of a renal failure. The okra extract significantly reduced the blood concentration of creatinin in comparison to diabetic control (Table 15). This was in accordance with previous works (48, 56). Danish et al. (56) reported that daily administration of *Albizzia lebbeck* stem bark extracts to diabetic rats for 45 days induced a significant decrease in the serum creatinin concentration.

**Table 15:** Serum and urine concentrations of creatinin of the treated rats.

	**Serum**	**Urine**
**Creatinin concentrations (**μ**mole/L)**		
AB	2.53 ± 0.10^a^	172.61 ± 1.17^d^
Met	2.47 ± 0.30^a^	156.11 ± 9.25^c^
T+	2.88 ± 0^b^	41.96 ± 2.40^a^
T–	2.38 ± 0.10^a^	122.69 ± 7.82^b^

AB, *Abelmoschus esculentus*; Met, Metformin; T+, Diabetic control; T–, Normal control. a, b, c, and d: in the same column, values with different letters differ significantly (*p* < 0.05).

## Conclusion

The study aimed at evaluating the antidiabetic activity of an optimized polyphenolic rich extract obtained from okra seeds. RSM was used with MAE to determine the optimal conditions for extracting polyphenols from okra seeds. It was found out that the solvent/dry matter ratio, the power of the microwave apparatus, the quadratic effect of the time (X_1_X_1_) and the interaction between the solvent/dry matter ratio and the operating power (X_3_X_2_) significantly influenced (*p* < 0.05) the polyphenolic content of the extracts. RSM permitted to define the conditions for maximum extraction of polyphenols from okra (87.66 ± 3.33 mg of GAE/g) as: microwave power of 330 W, with a solvent ratio of 97.04/1 for 9.5 min. Optimization thus permitted to determine the conditions for extraction of at least two-fold the average maximum TPC reported up to date from okra seeds. The optimized extract exhibited powerful antioxidant capacities with an IC_50_ of 3.99 ± 0.15 μg/mL in DPPH scavenging assay. It also acted as a non-competitive inhibitor of porcine pancreatic amylase, and showed good antidiabetic capacities on streptozotocin induced diabetic rats.

## Data availability statement

The original contributions presented in the study are included in the article/supplementary material, further inquiries can be directed to the corresponding author.

## Author contributions

CW and DK conceived the work, collected seeds, carried out experimentations, analyzed and interpreted data, and wrote the article. DM collected the seeds, assisted in experimentations, and read the article. HW supervised the work and read the article. All authors have approved the final article.

## Conflict of interest

The authors declare that the research was conducted in the absence of any commercial or financial relationships that could be construed as a potential conflict of interest.

## Publisher's note

All claims expressed in this article are solely those of the authors and do not necessarily represent those of their affiliated organizations, or those of the publisher, the editors and the reviewers. Any product that may be evaluated in this article, or claim that may be made by its manufacturer, is not guaranteed or endorsed by the publisher.

## References

[1] Pereira ASP Banegas-Luna AJ Peña-García J Pérez-Sánchez H Apostolides Z. Evaluation of the anti-diabetic activity of some common herbs and spices: providing new insights with inverse virtual screening. Molecules. (2019) 24:4030. 10.3390/molecules24224030 31703341 PMC6891552

[2] Solayman M Ali Y Alam F Islam MA Alam N Khalil MI. Polyphenols: potential future arsenals in the treatment of diabetes. Curr Pharm Des. (2016) 22:549–65. 10.2174/1381612822666151125001111 26601968

[3] Ong E Oh C Tan J Foo S Leo C. Pressurized hot water extraction of okra seeds reveals antioxidant, antidiabetic and vasoprotective activities. Plants. (2021) 10:1645. 10.3390/plants10081645 34451690 PMC8399463

[4] Vinatoru M Mason T Calinescu I. Ultrasonically Assisted Extraction (UAE) and Microwave Assisted Extraction (MAE) of functional compounds from plant materials. Trends Analytical Chem. (2017) 97:159–78. 10.1016/j.trac.2017.09.002

[5] Chuyen HV Nguyen MH Roach PD Golding JB Parks SE. Microwave-assisted extraction and ultrasound-assisted extraction for recovering carotenoids from Gac peel and their effects on antioxidant capacity of the extracts. Food Sci Nutr. (2018) 6:189–96. 10.1002/fsn3.546 29387378 PMC5778220

[6] Amirabbasi S Elhamirad AH Saeediasl MR Armin M Ziaolhagh SHR. Optimization of polyphenolic compounds extraction methods from Okra stem. J Food Measurement Charact. (2020) 15:717–34. 10.1007/s11694-020-00641-8

[7] Sharma A Baluja Z. Therapeutic effects of Glycine max (soybean): A summary. Int. J. Pharm. Biosci. (2015) 2:22–7.

[8] Ali K Meredith A Thomas H. Techniques for analysis of plant phenolic compounds. Molecules. (2013) 18:2328–75. 10.3390/molecules18022328 23429347 PMC6270361

[9] Gharaati Jahromi S. Extraction techniques of phenolic compounds from plants. In: Plant Physiological Aspects of Phenolic Compounds. London: Interchopen (2019). 10.5772/intechopen.84705

[10] Felipe O. Recent advances and comparisons of conventional and alternative extraction techniques of phenolic compounds. J Food Sci Technol. (2020) 57:4299–315. 10.1007/s13197-020-04433-2 33087945 PMC7550548

[11] Geng S Liu Y Ma H Chen C. Extraction and antioxidant activity of phenolic compounds from okra flowers. Tropical J Pharma Res. (2015) 14:807–14. 10.4314/tjpr.v14i5.10

[12] Ibukunoluwa F Jong J Won Y. Effect of extraction conditions on ultrasonic-assisted extraction of polyphenolic compounds from okra (Abelmoschus esculentus L.) leaves. Korean J Food Preserv. (2020) 27:476–86. 10.11002/kjfp.2020.27.4.476

[13] Woumbo C Kuate D Klang M Womeni H. Valorization of *Glycine max* (Soybean) seed waste: optimization of themicrowave-Assisted Extraction (MAE) and characterization of polyphenols from soybean meal using Response Surface Methodology (RSM). J Chem. (2021) 2021 12:4869909. 10.1155/2021/4869909

[14] Naima R Hannache H Oumam M Sesbou A Charrier B Pizzi A. Green extraction process of tannins obtained from Moroccan Acacia mollissima barks by microwave: Modeling and optimization of the process using the response surface methodology RSM. Arabian J Chem. (2019) 12:2668–84. 10.1016/j.arabjc.2015.04.032

[15] Lim Y Eom S. Kiwifruit cultivar “Halla gold” functional component changes during preharvest fruit maturation and postharvest storage. Scientia Horticulturae;. (2018) 234:134–9. 10.1016/j.scienta.2018.02.036

[16] Padmaja A Prasad N. Pomegranate (*Punica granatum* L) peel extract as a source of natural antioxidant. J Food Sci Engineer. (2011) 1:171–83.

[17] Association of Official Analytical Chemists [AOAC]. Official Methods of Analysis of the AOAC. 15th Edition. Washington, DC. (1990).

[18] Koziol M. Afrosimetric estimation of threshold saponin concentration for bitterness in quinoa (*Chenopodium quinoa Willd*). J Sci Food Agricul. (1991) 54:211–9. 10.1002/jsfa.2740540206

[19] Pauwels J Van Ranst E Verloo M Mvondo Ze A. Manuel de Laboratoire de Pédologie - méthodes d'analyses de sols et de plantes?; equipment et gestion des stocks de verrerie et de produits chimiques. Publications Agricoles nr. 28, A.G.C.D., Bruxelles (1992). p. 180.

[20] Oyaizu M. Studies on products of browning reaction prepared from glucosamine. Japanese J Nutr. (1986) 44:307–15. 10.5264/eiyogakuzashi.44.307

[21] Mensor L Menezez F Leitao G. Screning of Brazilian plant extracts for antioxidant activity by the use of DPPH free radical method. Phytotherapy Res. (2001) 15:127–130. 10.1002/ptr.687 11268111

[22] Worthington V. Alpha-amylase. In:Worthington V, editor. Worthington enzyme manual freehold. New Jersey, NJ: Worthington Biochemical Corp (1993). pp. 36–41.

[23] OCDE. Essai n° 425: Toxicité aiguë par voie orale: méthode de l'ajustement des doses, Lignes directrices de l'OCDE pour les essais de produits chimiques, Section 4, Éditions OCDE, Paris (2022). 10.1787/9789264071056-fr

[24] Friedewald W Levy R Fredrickson D. Estimation of the concentration of low-density lipoprotein cholesterol in plasma, without use of the preparative ultracentrifuge. Clin Chem. (1972) 18:499–502. 10.1093/clinchem/18.6.499 4337382

[25] Yagi K. A simple fluorometric assay for lipoperoxide in blood plasma. Biochemistry Res. (1976) 15:212–6. 10.1016/0006-2944(76)90049-1 962904

[26] Ellman G. Tissue sulfhydryl groups. Arch Biochem Biophys. (1959) 82:70–7. 10.1016/0003-9861(59)90090-6 13650640

[27] Peter E Nagendrappa P Ajayi C Sesaazi C. Total polyphenols and antihyperglycemic activity of aqueous fruits extract of *Abelmoschus esculentus*: modeling and optimization of extraction conditions. PLoS ONE. (2021) 16:e0250405 10.1371/journal.pone.0250405 33861803 PMC8051779

[28] Hu L Yu W Li YN Tang Z. Antioxidant activity of extract and its major constituents from okra seed on rat hepatocytes injured by carbon tetrachloride. BioMed Res Int. (2014) 341291:9. 10.1155/2014/341291 24719856 PMC3955651

[29] Sanja Bogdan N Nevena L Jelena J Andrea S Nataša Š. The impact of high-power ultrasound and microwave on the phenolic acid profile and antioxidant activity of the extract from yellow soybean seeds. Industrial Crops Product. (2018) 122:223–31. 10.1016/j.indcrop.2018.05.078

[30] Xuan T Vo L Tran Q Long G Tran T Pham T. Extraction process of polyphenols from soybean (*glycine max* l.) sprouts: optimization and evaluation of antioxidant activity. Processes. (2019) 7:489. 10.3390/pr7080489

[31] Mohamed A Chang Y. Optimization of phenolics and dietary fibre extraction from date seeds. Food Chemistry. (2008) 108:977–85. 10.1016/j.foodchem.2007.12.009 26065761

[32] Joglekar A May A. Product excellence through design of experiments. Cereal Food World. (1987) 32:857–68.

[33] Graham J Agbenorhevi J Kpodo F. Total Phenol content and antioxidant activity of Okra seeds from different genotypes. Am J Food Nutr. (2017) 5:90–4. 10.12691/ajfn-5-3-2

[34] Oliveira R Mancini M Oliveira F Passos T Quilty B Thiré R. FTIR analysis and quantification of phenols and flavonoids of five commercially available plants extracts used in wound healing. Matéria (Rio de Janeiro). (2016) 21:767–79. 10.1590/S1517-707620160003.0072

[35] Kozłowicz K Rózyło R Gładyszewska B Matwijczuk A Gładyszewski G Chocyk D. Identification of sugars and phenolic compounds in honey powders with the use of GC–MS, FTIR spectroscopy, and X-ray diffraction. Sci Rep. (2020) 10:1–10. 10.1038/s41598-020-73306-7 33004933 PMC7529813

[36] Sulistyowati E Martono S Riyanto S Lukitaningsih E Rohman A. Rapid quantitative analysis of daidzein and genistein in soybeans (Glycine max (L). *Merr*) using FTIR spectroscopy and multivariate calibration. J Applied Pharm Sci. (2020) 10:117–23.

[37] Doreddula SK Bonam SR Gaddam DP Desu BS Ramarao N Pandy V. (2014). Phytochemical analysis, antioxidant, antistress, and nootropic activities of aqueous and methanolic seed extracts of ladies finger (*Abelmoschus esculentus* L.) in Mice. Sci World J. (2014) 14:519848. 10.1155/2014/519848 25401145 PMC4221879

[38] Nandiyanto ABD Oktiani R Ragadhita R. How to read and interpret FTIR spectroscope of organic material. Indones J Sci Technol. (2019) 4:97–118.

[39] Baker M Trevisan J Bassan P Bhargava R Butler HJ Dorling K. Using Fourier transform IR spectroscopy to analyze biological materials. Nat Protoc. (2014) 9:1771–91. 10.1038/nprot.2014.110 24992094 PMC4480339

[40] Kumar J Prasad A. Identification and comparison of biomolecules in medicinal plants of Tephrosia tinctoria and Atylosia albicans by using FTIR. Romanian J Biophysics. (2011) 21:63–71.

[41] Pharmawati M Wrasiati L. Phytochemical screening and FTIR spectroscopy on crude sextract from Enhalus acoroides leaves. Malaysian J Analytical Sci. (2020) 24:70–7.

[42] Ortaç D Cemek M Karaca T Büyükokuroglu ME Özdemir ZÖ Kocaman AT. *In vivo* anti-ulcerogenic effect of okra (Abelmoschus esculentus) on ethanol-induced acute gastric mucosal lesions. Pharmaceutical Biol. (2018) 56:165–75. 10.1080/13880209.2018.1442481 29513129 PMC6130549

[43] Uddin Z., Khatun, J., and Khan, M. M., and Haque, M. Evaluation of in vitro antioxidant activity of okra mucilage and its antidiabetic and antihyperlipidemic effect in alloxan-induced diabetic mice. Food Sci Nutr. (2021) 9:6854–65. 10.1002/fsn3.2641 34925813 PMC8645766

[44] Quan NV Xuan TD Tran HD Thuy NTD Trang LT Huong CT. Antioxidant, α-amylase and α-glucosidaseinhibitory activities and potential constituents of *Canarium tramdenum* bark. Molecules. (2019) 24:605. 10.3390/molecules24030605 30744084 PMC6385046

[45] Dhital S Gidley J Warren J. Inhibition of -amylase activity by cellulose: kinetic analysis and nutritional implications. Carbohydrate Polymers. (2015) 123:305–12. 10.1016/j.carbpol.2015.01.039 25843863

[46] Nsor-Atindana J Yu M Goff H Chen M Zhong F. Analysis of kinetic parameters and mechanisms of nanocrystalline cellulose inhibition of (-amylase and (-glucosidase in simulated digestion of starch. Food Function. (2020) 34:D0FO00317D. 10.1039/D0FO00317D 32412562

[47] Kawser H Abdal D Han J Yin Y Kim K Kumar Saha S. Molecular mechanisms of the anti-obesity and anti-diabetic properties of flavonoids. Int J Mol Sci. (2016) 17:569. 10.3390/ijms17040569 27092490 PMC4849025

[48] Petchi RR Vijaya C Parasuraman S. Antidiabetic activity of polyherbal formulation in streptozotocin—nicotinamide induced diabetic wistar rats. J Tradit Complement Med. (2014) 4:108-17. 10.4103/2225-4110.126174 24860734 PMC4003700

[49] Gupta R Sharma SB Singh UR. Salutary effects of germinated glycine max seeds on post prandial hyperglycemia and dyslipidemia - evidence from in-vivo and in-vitro studies. Altern Integr Med. (2017) 6:237. 10.4172/2327-5162.1000237

[50] Adams DM Yakubu MT. Aqueous extract of Digitaria exilis grains ameliorates diabetes in streptozotocin-induced diabetic male Wistar rats. J Ethnopharmacol. (2019) 249:112383. 10.1016/j.jep.2019.112383 31733308

[51] Weickert M Pfeiffer A. Metabolic effects of dietary fiber consumption and prevention of diabetes. J Nutr. (2008) 138:439–42. 10.1093/jn/138.3.439 18287346

[52] Paquet E. Etude de la stabilité des fibres alimentaires seules et en mélange dans un breuvage à base de suspensions de fruits et compréhension de leur effet sur les réponses glycémique et insulinémique. Mémoire, université Laval, France (2010). p. 147

[53] Mohammed A Koorbanally NA Islam MS. Anti-diabetic effect of Xylopia aethiopica (Dunal) A. Rich. (Annonaceae) fruit acetone fraction in a type 2 diabetes model of rats. J. Ethnopharmacol. (2016) 180:131–9. 26795545 10.1016/j.jep.2016.01.009

[54] Emeka A Uzoma A. Mechanisms of actions of some bioactive anti-diabetic principles from phytochemicals of medicinal plants: a review. Indian J Nat Products Res. (2018) 9:85–96.

[55] Esmaeilzadeh D Razavi BM Hosseinzadeh H. Effect of Abelmoschus esculentus (okra) on metabolic syndrome: a review. Phytotherapy Res. (2020) 34:2192–202. 10.1002/ptr.6679 32222004

[56] Danish A Vikas Kumar A Pushpraj S Hemant K Vishal D Vatsala M. Antidiabetic, renal/hepatic/pancreas/cardiac protective and antioxidant potential of methanol/dichloromethane extract of Albizzia lebbeck benth. stem bark (ALEx) on streptozotocin induced diabetic rats. BMC. (2014) 14:1–17. 10.1186/1472-6882-14-243 25026962 PMC4223618

[57] Brijyog A Shish S Sushil K Shweta V. Antidiabetic activity of newly formulated oral polyherbal tablets in alloxan induced diabetic rats. J Clin Toxicol. (2019) 9:1–5.

[58] Abd El Latif M Mohamed N Zaki N Abbas M Sobhy H. Effects of Soybean isoflavone on lipid profiles and antioxidant enzyme activity in Streptozotocin induced diabetic rats. Global J Pharmacol. (2014)8:378–84. 10.5829/idosi.gjp.2014.8.3.111620016722

[59] Ma M Mu T. Anti-diabetic effects of soluble and insoluble dietary fibre from deoiled cumin in low-dose streptozotocin and high glucose-fat diet-induced type2 diabetic rats. J Funct Foods. (2015) 5:186–96. 10.1016/j.jff.2016.05.011

[60] Fagbohun OF Awoniran PO Babalola OO Agboola FK Msagati TAM. Changes in the biochemical, hematological and histopathological parameters in STZ-Induced diabetic rats and the ameliorative effect of *Kigelia africana* fruit extract. Heliyon. (2020) 6:13. 10.1016/j.heliyon.2020.e03989 32462092 PMC7243140

[61] Nambirajan G Karunanidhi K Ganesan A Rajendran R Kandasamy R Elangovan A. Evaluation of antidiabetic activity of bud and flower of *Avaram Senna* (*Cassia auriculata* L.) In high fat diet and streptozotocin induced diabetic rats. Biomed Pharmacother. (2018) 108:1495–506. 10.1016/j.biopha.2018.10.007 30372851

[62] Dubey S Yadav C Bajpeyee A Singh MP. Effect of *Pleurotus fossulatus* aqueous extract on biochemical properties of liver and kidney in streptozotocin-induced diabetic rat. Diabetes Metab Syndr Obes. (2020) 13:3035–46. 10.2147/DMSO.S265798 32904440 PMC7455752

[63] Al-Jaghthmi A Zeid A. Hypoglycemic and hepatoprotectve effect of rhizophora mucronata and avicennia marina against streptozotocin-induced diabetes in male rats. J Advanced Vet Animal Res. (2020) 7:177–85. 10.5455/javar.2020.g408 32219125 PMC7096112

